# Preparation, In Vitro Characterization, and Cytotoxicity Evaluation of Polymeric pH-Responsive Hydrogels for Controlled Drug Release

**DOI:** 10.3390/pharmaceutics14091864

**Published:** 2022-09-03

**Authors:** Muhammad Suhail, Jia-Yu Liu, Ming-Chia Hung, I-Hui Chiu, Muhammad Usman Minhas, Pao-Chu Wu

**Affiliations:** 1 School of Pharmacy, Kaohsiung Medical University, 100 Shih-Chuan 1st Road, Kaohsiung 80708, Taiwan; 2 College of Pharmacy, University of Sargodha, Sargodha 40100, Pakistan; 3 Department of Medical Research, Kaohsiung Medical University Hospital, Kaohsiung 80708, Taiwan; 4 Drug Development and Value Creation Research Center, Kaohsiung Medical University, Kaohsiung 80708, Taiwan

**Keywords:** hydrogels, swelling, in vitro drug release, biodegradation, cytotoxicity study

## Abstract

The aim of the current investigation was based on the development of pH-responsive hydrogels of chondroitin sulfate, carbopol, and polyvinyl alcohol polymerized with acrylic acid in the presence of ammonium persulfate and ethylene glycol dimethylacrylate for controlled drug delivery. A free radical polymerization technique was used for the preparation of these pH-responsive hydrogels. The gel fraction of the prepared hydrogels was increased with the increase in the chondroitin sulfate, carbopol, polyvinyl alcohol, and acrylic acid content, while the sol-fraction was decreased. Swelling and drug release studies were performed in various pH conditions. Greater swelling and drug release were observed at high pH values (pH 4.6 and 7.4) as compared to low pH value (pH 1.2), representing the pH-responsive nature of the synthesized hydrogels. Porosity and drug loading were increased with the incorporation of high concentrations of hydrogel contents except polyvinyl alcohol, which showed reverse effects. Similarly, biodegradation study reported a slow degradation rate of the prepared hydrogels with the increase in hydrogel constituents. Cytotoxicity study proved the safe use of developed hydrogels as no toxic effect was shown on T84 human colon cancer cells. Similarly, various characterizations, including Fourier transform infrared spectroscopy, thermogravimetric analysis, differential scanning calorimetry, X-ray diffraction, and scanning electron microscopy, were performed for prepared hydrogels. Hence, we could demonstrate that the prepared hydrogels can be used as a promising drug carrier for the controlled delivery of drugs.

## 1. Introduction

Non-steroidal anti-inflammatory drugs (NSAIDs) are considered the most suitable candidates for the synthesis of controlled release preparations, especially through the oral route. However, several authors have reported severe adverse effects of NSAIDs on the gastric mucosa. Due to short biological half-lives, multiple administrations of NSAIDs are required daily [1]. Diclofenac sodium (DS) is a potent non-steroidal anti-inflammatory drug with pronounced analgesic properties. It is recommended for the long-term management of osteoarthritis, rheumatoid arthritis, and ankylosing spondylitis. The reported biological half-life of DS is 60–120 min [2]. Due to multiple intakes of DS, certain severe side effects, including ulceration, bleeding, or perforation of intestinal wall, are commonly observed [3]. Due to its short biological half-life, multiple intakes, and associated adverse effects, DS is considered as an ideal candidate for controlled drug delivery. Hence, due to good biocompatibility, biodegradability, stability, and low toxicity, hydrogels are considered as a one of the most promising carriers for the controlled delivery of therapeutic agents [4,5].

Hydrogels are three-dimensional polymeric networks that have the capability to convey and release drugs on targeted sites [6]. Hydrogels are such materials that not only absorb a high quantity of water but also retain their integrity within water for a long period of time [7,8,9,10,11,12,13]. Hydrogels are valuable due to their ease of preparation, small size, mouldability, immunity to electromagnetic radiation, biodegradability, and biocompatibility [14]. Due to these unique properties, researchers have been taking great interest in the preparation of especially stimuli-responsive hydrogels for the last couple of decades [15]. Due to different stimuli, such as pH, electromagnetic radiation, temperature, ionic strength, and nature of swelling reagents, the responsive nature of stimuli-sensitive hydrogels is exhibited. Swelling at various pH values is shown by pH-responsive hydrogels due to the presence of acidic or basic pendent groups. Water absorbing and swelling capability of hydrogels are dependent on hydrophilic groups, including CONH_2_, OH, SO_3_H, and CONH [16]. Hydrogels protect the proteins, drugs, and peptides from unfriendly environments [17]. Therefore, due to such beneficial characteristics, hydrogels can be employed in oral delivery of therapeutic agents [18].

Chondroitin sulfate (CS) is the main structural component in connective tissues and cartilages. A compressive strength is given by CS to connective tissues by regulating its water content. CS has unique characteristics, including absorption of water in high quantity, biodegradability, and multifunctionality, which are suitable for different bio-applications. Furthermore, the existence of active functional groups, such as COO and SO_3_, provides access to biological functionalities, currently exploited in in vivo cartilage repair applications [19]. Carbopol-934 (CP) is a high-molecular-weight synthetic polymer. It consists of long chains of acrylic acid, which is employed commonly for the preparation of drug delivery systems because of its high biodegradability, aqueous-solubility, and bio-adhesiveness [20]. Polyvinyl alcohol (PVA) is also one of the most commonly used synthetic polymers in biomedical and pharmaceutical fields. Furthermore, due to high mechanical characteristics, PVA supports cell adhesion, propagation, and migration [21]. Acrylic acid (Aa)-based hydrogels have unique features, such as high hydrophilicity and stimuli-responsive behavior, due to the presence of a high number of carboxyl groups. These groups lead to ionization, and, as a result, polymer chains are relaxed. Thus, maximum swelling of hydrogel is achieved. The swelling and de-swelling of Aa-based hydrogels are dependent on the pH and ionic strength of the environment; thus, they are used widely in microdevices and sensors. Furthermore, Aa is commonly used as mucoadhesives for delivery of drugs and surface coatings for biomedical devices because of its excellent biocompatibility [22].

The novelty of the present investigation is based on the incorporation of the pH-sensitive reagents, which enabled the prepared hydrogel to sustain the release of the DS for an extended period of time. The developed hydrogel shows low swelling and drug release at pH 1.2 while high at 4.6 and especially at pH 7.4, thus not only protecting the gastrointestinal tract (GI) from the severe adverse effects of the drug but also keeping the drug away from the acidic environment of the stomach. Due to the incorporation of both natural and synthetic polymers, the safety and mechanical strength of the fabricated hydrogel are increased, as shown by cytotoxicity and biodegradation studies. Thus, the results show that the novel prepared pH-responsive network of hydrogel could be considered as a suitable drug carrier for the controlled release of drugs.

## 2. Materials and Methods

### 2.1. Materials

Ethylene glycol dimethylacrylate (EGDMA) (purity = 98%) and DS were purchased from Alfa-Aesar (Ward Hill, MA, USA). CS (purity = 90%) was acquired from Sigma-Aldrich (St. Louis, MO, USA). Ammonium persulfate (APS) (purity = 98%) and Aa (purity = 98% extra pure) were obtained from Showa (Osaka, Japan) and Acros (Carlsbad, CA, USA), respectively. PVA (MW = 130,000, 99+% hydrolyzed) was procured from Sigma-Aldrich (Chemie Gmbh, Riedstr, Steinheim, Germany), while CP (purity = 99%) was obtained from Noveon, Inc (Cleveland, OH, USA).

### 2.2. Synthesis of CS/CP/PVAcPAa Hydrogels

**Figure 1:**
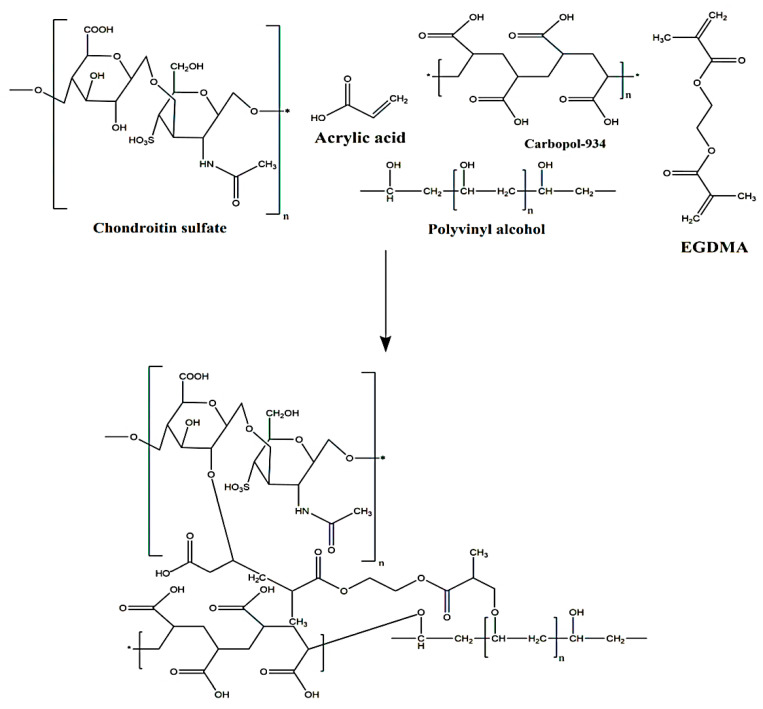
Proposed chemical structure of CS/CP/PVAcPAa hydrogel.

Various feed ratios of CS, CP, PVA, and Aa were crosslinked by EGDMA in the presence of APS for the development of chondroitin sulfate/carbopo-934/polyvinyl alcohol-co-poly(acrylic acid) (CS/CP/PVAcPAa) hydrogels via a free radical polymerization technique. Hence, weighed quantity of CS, CP, and PVA was taken and dissolved in a specific volume of deionized distilled water at a temperature of 50 and 80 °C with 50 rpm. Similarly, APS was dissolved in deionized distilled water. Aa and EGDMA were already available in solution form. Initially, CP solution was poured into CS solution. APS solution was added slowly into the PVA solution, which was added later into the mixture of CS and CP. Aa was added dropwise into the polymers mixture. Finally, EGDMA was poured into the mixture of polymers and monomer. The whole mixture was kept on stirring for 15 min. A transparent solution was formed, which was purged by a nitrogen gas to remove any dissolved oxygen. The solution was transferred into the glass molds, which were placed in water bath at 55 °C for initial 2 h. The temperature was increased from 55 to 60 °C for the next 24 h. The prepared gel was cut into 6 mm and 8 mm discs, respectively. The prepared discs of gel were washed by a mixture of water and ethanol (50:50 *v*/*v*) in order to remove any attached impurity to the surface of the discs. The discs were subjected to atmospheric temperature for 24 h. After that, the discs were placed in a vacuum oven at 40 °C for complete dryness. The various combinations of polymers and monomer for the various formulations of prepared hydrogels are given in Table 1, while proposed chemical structure is shown in Figure 1.

### 2.3. Fourier Transform Infrared Spectroscopy (FTIR)

FTIR spectra of CS, CP, PVA, Aa, DS, unloaded, and drug-loaded hydrogels were recorded. Initially, all samples were crushed and grounded thoroughly by using pestle and mortar. Attenuated total reflectance FTIR (Thermo Fisher Scientific, Ishioka, Japan) was employed for spectral analysis in the range of 4000–500 cm^−1^ [23].

### 2.4. Thermogravimetric Analysis (TGA)

The thermal stability of hydrogel constituents and fabricated hydrogels was investigated by TGA (PerkinElmer Simultaneous Thermal Analyzer STA 8000). Grounded samples (0.5 to 5 mg) were placed in a platinum pan, which was attached with a microbalance. TGA was operated within temperature range of 40–600 °C with a flow rate of 20 °C/min under constant flow of nitrogen gas [24].

### 2.5. Differential Scanning Calorimetry (DSC)

The fusion heat of CS, CP, PVA, and CS/CP/PVAcPAa hydrogel was measured by DSC (PerkinElmer DSC 4000) analysis. Samples (0.5 to 5 mg) were taken and placed in a platinum pan connected with microbalance. Scanning of all samples was carried out from 50–400 °C with a heating rate of 20 °C/min under dry nitrogen flow of 20 mL/min [25].

### 2.6. X-ray Diffraction Studies

The crystallinity of CS, CP, PVA, DS, unloaded, and drug-loaded CS/CP/PVAcPAa hydrogel was evaluated by XRD ((XRD-6000, Shimadzu, Tokyo, Japan). A specific amount of sample was taken and placed in plastic sample holder. A flat glass slide was used for smoothing the surface of sample. XRD analysis was performed within the range of 10–60° with an angle of 2θ 2°/min [26].

### 2.7. Scanning Electron Microscopy (SEM)

The surface configuration of the developed polymeric hydrogel was examined by SEM (JSM-5300 model, JEOL, Tokyo, Japan). The sample of hydrogel was mounted on aluminum point by the help of sticky tape. A layer of gold was coated with the aid of gold sputter coater in an inert environment using vacuum evaporator. Photomicrographs were captured, and, thus, the surface morphology was analyzed [27].

### 2.8. Sol–Gel Fraction

Sol and gel fraction was used to estimate the fraction of reactants consumed in the development of hydrogels. Hence, a weighed dried disc of hydrogel was placed in Soxhlet apparatus for extraction process while using boiling water. The extraction process was carried out for 12 h. After that, the extracted disc of hydrogel was taken out and placed in a vacuum oven at 40 °C for complete dryness. The dried extracted disc was weighed again [28]. Sol and gel fraction was estimated by using the given formula:

(1) $$\mathrm{Sol}\;\mathrm{fraction}\;\%=\frac{{\;H}_{1}-{\;H}_{2}}{{\;H}_{1}}\times 100$$

(2) Gel fraction=100−Sol fraction

H_1_ represents the initial weight of the dried disc of the hydrogel, while H_2_ indicates the final weight after the extraction process.

### 2.9. Porosity Study

The penetration ability of the fluid through the CS/CP/PVAcPAa hydrogel was determined by porosity study. Hence, solvent displacement method was performed in order to determine the porosity of the prepared hydrogels. Absolute ethanol was used as a displacement solvent. Dried disc of accurate weight of the hydrogel (M_1_) was immersed in absolute ethanol for 72 h. After achieving equilibrium swelling, disc was taken out, blotted with filter paper, and then weighed again (M_2_) on weighing balance [29]. Porosity percent of all formulations was determined by the given formula:

(3) $$(\%)\;\mathrm{Porosity}=\frac{M_{2}-M_{1}}{\rho V}\times 100$$

ρ indicates the density of absolute ethanol and V represents the swelling volume of hydrogel discs.

### 2.10. Biodegradation Study

The biodegradation of prepared hydrogels was carried out in phosphate buffer solution of pH 7.4 at body temperature °C. Hence, hydrogel formulation of precise weight was immersed in a buffer solution of pH 7.4 at various intervals of time, i.e., 1, 3, 5, 7, 10, and 14 days. After that, the hydrogel formulation was removed and placed in a vacuum oven at 40 °C for dryness [30]. Biodegradation rate of the hydrogel formulation was estimated by the given formula:

(4) $$Z=\frac{A_{1}-A_{2}}{A_{1}}$$

Z represents the degradation; A_1_ indicates the weight of dried hydrogel formulation; while A_2_ is the weight of hydrogel formulation after immersion at time (t).

### 2.11. Swelling Study

The swelling of developed hydrogel was investigated in three different pH values, i.e., pH 1.2, 4.6, and 7.4, at body temperature. Hence, dried hydrogel samples of accurate weight were immersed in the respective buffer solutions. After specific time intervals, the samples were removed, blotted with filter paper, and weighed again. This process was continued until an equilibrium weight of hydrogel was achieved [31]. Swelling of hydrogel was determined by the given formula:

(5) $$\left(q\right)=\frac{{\;K}_{2}}{K_{1}}$$

q shows the dynamic swelling, K_1_ represents the initial weight of dried disc of the hydrogel before swelling, whereas K_2_ reveals the final weight after swelling at time t.

### 2.12. Polymer Volume Fraction

The fraction of polymer of the fabricated hydrogel in swelled state was determined by polymer volume fraction. It is represented by K. Equilibrium volume swelling (Veq) data of the fabricated hydrogel were employed for the estimation of the polymer volume fraction in pH 1.2, 4.6, and 7.4, respectively [32]. Hence, polymer volume fraction was estimated by the given formula:

(6) $$K=\frac{1}{\mathrm{Veq}}$$

### 2.13. Drug Loading

Drug loading study was carried out for all formulations of CS/CP/PVAcPAa hydrogel by diffusion method. Therefore, weighed dried hydrogel formulations were submerged in 1% DS solution of phosphate buffer pH 7.4 for 5 days. After that, the hydrogel formulations were taken out after attaining equilibrium swelling and loading. The surface entrapped drug was removed by washing the hydrogel formulations with deionized distilled water. The loaded hydrogel formulations were placed at 40 °C in a vacuum oven till dried completely.

The estimation of loaded drug by the hydrogel formulation was determined by extraction and weighed methods. In extraction method, a 25 mL phosphate buffer solution of pH 7.4 was taken within weighed loaded hydrogel formulations that were immersed. Samples were collected after specific time intervals and medium was replenished by same fresh medium. This act was continued till the entire drug was removed completely from the loaded formulations of hydrogel. After that, the collected samples were analyzed by using UV–Vis spectrophotometer (U-5100, 3J2-0014, Tokyo, Japan) at λ_max_ 260 nm in triplicate.

Weight method is another method used for the determination of drug-loaded contents by the developed hydrogel. Thus, in this procedure, the weight of unloaded hydrogel formulation was subtracted from the weight of loaded hydrogel formulation [33]. The determination of loaded drug by the developed hydrogel formulation was performed by the given formula:

Drug-loaded = P_L_ − P_UL_ (7)

P_L_ shows the weight of drug-loaded hydrogel formulation, whereas P_UL_ represents the weight of unloaded hydrogel formulation [34].

### 2.14. Drug Release Studies

The pH-dependent release of drug from the developed hydrogels and commercial product Cataflam (25 mg, Novartis, Basel, Switzerland) was investigated in three various buffer solutions of pH 1.2, 4.6, and 7.4, respectively. Previously, Cataflam and weighed drug-loaded formulations of hydrogel were immersed in 900 mL respective buffer solutions while using a USP dissolution apparatus type II (Sr8plus Dissolution Test Station, Hanson Research, Chatsworth, CA, USA) at 37 ± 0.5 °C. Aliquots of 5 mL were withdrawn periodically and replenished with same fresh buffer solution of the same concentration to maintain the sink conditions. Collected samples were filtered, diluted, and then analyzed on UV–Vis spectrophotometer (U-5100, 3J2-0014, Tokyo, Japan) in triplicate at λ_max_ 260 nm [35].

### 2.15. Kinetic Modeling

Different kinetic models, including zero-order kinetics, first-order kinetics, Higuchi model, and Korsmeyer–Peppas model, were used for the determination of drug release mechanism and other various parameters of the developed hydrogel. Drug release data of the developed hydrogel were used for the interpretation of order and mechanism of drug from the fabricated hydrogel [36].

### 2.16. Cytotoxicity Study

The cytotoxicity evaluation of the CS/CP/PVAcPAa hydrogel was analyzed by a Cell Counting Kit-8 (CCK-8) assay. Hence, inoculation of T84 (human colon cancer cells) cells was performed in 96-well plates (1 × 10^5^ cells/well) at body temperature. After 24 h, medium was replaced by fresh cell cultural medium comprising hydrogel formulation within a concentration range of 5–40 mg/mL. After that, addition of 10 μL of CCK8 solution per well was carried out and then plate incubation was performed again for 2 h at 37 °C. Finally, absorbance was detected at 450 nm while using microplate Spectrophotometer (Epoch, BioTek, Winooski, VT, USA) [37]. Cell viability was determined by the given formula:

(OD_sample_ − OD_Blank_)/(OD_control_ − OD_Blank_) × 100 (8)

### 2.17. Statistical Analysis

SPSS Statistic software 22.0 (IBM Corp, Armonk, NY, USA was employed for the Statistical analysis. The difference between the experiments was determined by using Student’s *t*-test. The results obtained were statistically considered significant because of the *p*-value, which was found less than 0.05.

## 3. Results and Discussion

### 3.1. Synthesis of CS/CP/PVAcPAa Hydrogels

**Figure 2:**
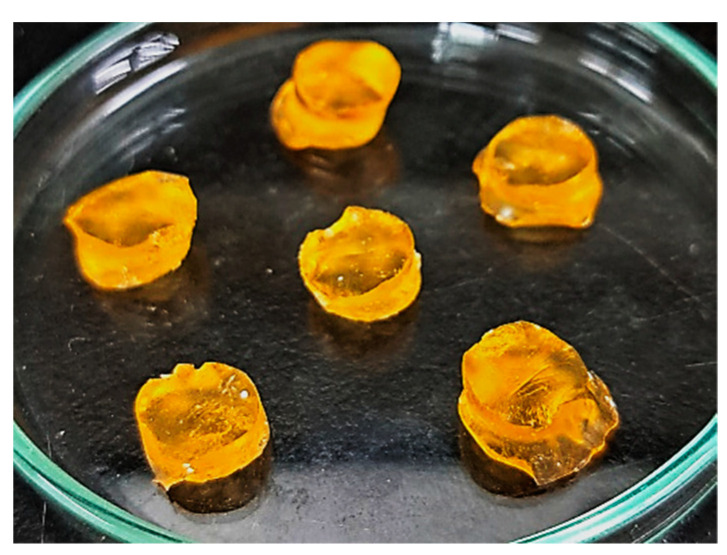
Physical appearance of CS/CP/PVAcPAa hydrogel.

A series of CS/CP/PVAcPAa hydrogel formulations were prepared by the free radical polymerization technique. The main purpose of incorporation of the various concentrations of CS, CP, and PVA with Aa was to evaluate the effects of the reagents on developed hydrogel systems. Hence, a number of studies were carried out for the developed hydrogels. Due to the usage of pH-sensitive polymers and monomer, the pH-sensitivity of the fabricated hydrogel was increased, and, as a result, maximum swelling and drug release were achieved at a high pH value. The pH-sensitive prepared hydrogels not only sustained the release of the DS but also protected the stomach from the adverse effects of the DS. The physical appearance of the prepared hydrogel is shown in Figure 2.

### 3.2. FTIR

**Figure 3:**
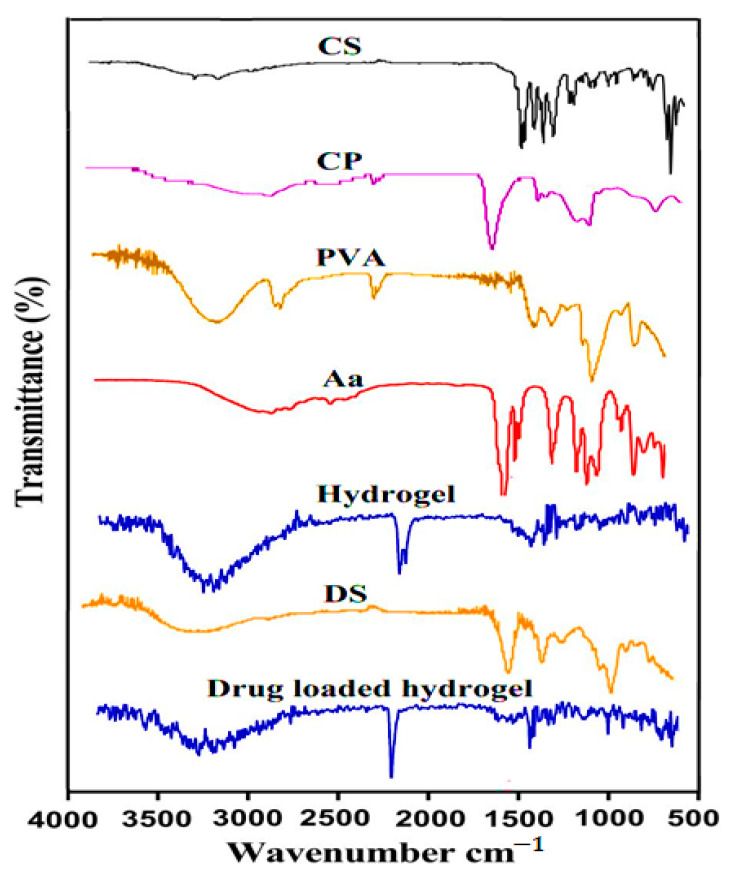
FTIR spectra of CS, CP, PVA, Aa, the unloaded hydrogel, DS, and the drug-loaded hydrogel.

The structural arrangements of hydrogel ingredients and its formulation were evaluated by FTIR analysis as indicated in Figure 3. The FTIR spectra of CS indicated the existence of OH and N–H stretching by a peak at 3351 cm^−1^, where the N–H stretching overlapped the OH stretching. An amide group was observed at 1612 cm^−1^, while bands at 1369 and 1408 were assigned to stretching vibration of O–H and C–O, indicating the existence of a carboxyl group. The stretching vibration of S=O groups was depicted by a peak at 1230, which is a prominent band of CS. Similarly, the stretching vibration of C–O was observed at 1032 cm^−1^. Same peaks of CS were observed by Crispim and co-workers [38], which further supports our finding. The FTIR spectra of CP presented stretching vibrations of OH and C=O by peaks at 2570 and 1690 cm^−1^, whereas the band at 2932 cm^−1^ indicated R-CH_2_ stretching vibration, respectively [39]. Likewise, PVA presented its FTIR spectra by peaks at 3398 cm^−1^ and 2962, revealing the existence of O-H and –CH_2_ group stretching vibrations. Deformation of –OH was observed by a band at 1449 cm^−1^ [40]. Aa indicated its FTIR spectra by peaks at 2969 and 1712, which were attributed to stretching vibrations of –CH_2_– and carboxyl group. C=O and C–C stretching were confirmed by peaks at 1642 and 1304 cm^−1^, respectively. Similarly, a band at 1168 cm^−1^ indicated the stretching vibration of C–O [41]. The FTIR spectra of CS/CP/PVAcPAa hydrogel confirmed the presence of various peaks of the polymers and monomer with altered intensity. The prominent peaks of CS at 1369, 1612 cm^−1^ and CP at 1690, 2932 cm^−1^ were moved to 1382, 1590, 1650, and 2950 cm^−1^ peaks of the developed hydrogel. Similarly, distinct peaks of PVA and Aa were changed from 1449, 3398 cm^−1^ and 1304, 1642 cm^−1^ to 1480, 3408, 1345, and 1612 cm^−1^ peaks of the fabricated hydrogel. This all indicated the synthesis of the polymeric network of the hydrogel. The FTIR spectra of DS are shown in Figure 3. DS indicated a distinctive band at 3338 cm^−1^, revealing the stretching vibration of COOH. Similarly, peaks at 1612 and 3412 cm^−1^ indicated C=C and N–H stretching vibrations [42]. Certain peaks of DS were changed slightly in loaded hydrogel formulation due to encapsulation of DS by the fabricated network of hydrogel, as indicated in Figure 3. The distinct peaks of DS were changed from 1612 and 3414 cm^−1^ to 1590 and 3430 cm^−1^ peaks of the loaded hydrogel formulation. This all indicated the successful loading of DS by the developed hydrogel without any interaction with hydrogel contents [43].

### 3.3. TGA

**Figure 4:**
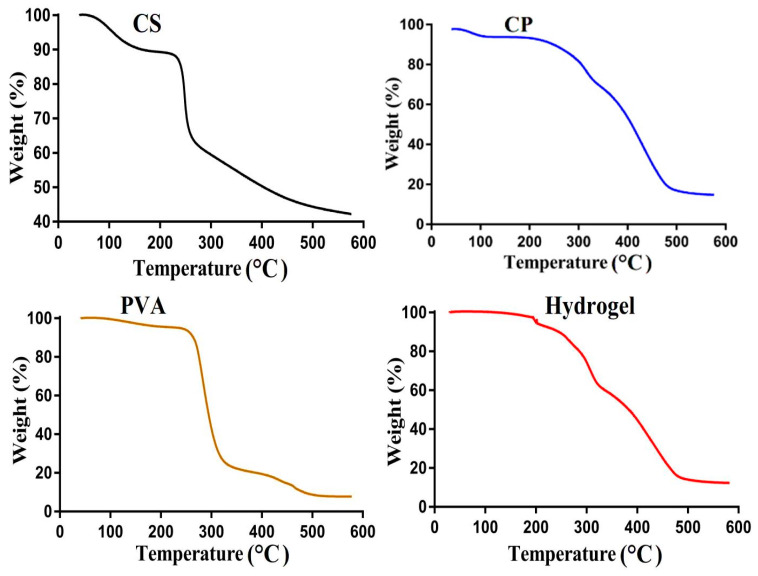
TGA of CS, CP, PVA, and CS/CP/PVAcPAa hydrogel.

TGA was performed for CS, CP, PVA, and the developed hydrogel to know their thermal stability with the increase in the temperature, as shown in Figure 4. The weight loss of CS occurred dramatically in three different stages. Initially, weight loss of 8% was detected during the first stage within a temperature range of 80–205 °C. During the second stage, further weight loss of 37% was detected as the temperature approached 260 °C. At the third stage, a loss of 18% in weight was observed at 450 °C. Further increase in temperature led to the entire degradation of CS. The weight loss of CS may be correlated with the degradation of carboxylate and sulfonate functional groups [44]. The TGA of CP presented a weight loss of 5% with the increase in temperature up to 250 °C during the first stage. Similarly, 18% and 54% weight loss was detected at temperatures of 305 and 480 °C during the second and third stage of degradation [45]. In the case of PVA TGA, initially, a negligible loss of 4% in weight was detected at 250 °C during the first stage. An increase in temperature up to 303 and 470 °C resulted in weight loss of 62% and 10%, respectively, during the second and third stages of degradation [46]. Similarly, weight loss of the developed hydrogel occurred in three different stages. Initially, during the first stage of degradation, 3% loss in weight was detected as the temperature reached 200 °C. Further degradation of 27% in the weight of the prepared hydrogel was observed as the temperature approached 303 °C during the second stage. Finally, a weight loss of 42% was observed at 495 °C during the third stage, and, after that, entire degradation started, which may be correlated with the decompositions of functional groups of polymers. Comparing the thermal stability of the polymers with the prepared hydrogel, we can see that the thermal stability of the developed hydrogel was higher than its unreacted polymers contents. This increase in thermal stability of the hydrogel is basically increased in the thermal stability of CS, CP, and PVA after the crosslinking and polymerization process. Barkat and coworkers prepared pH-responsive polyethylene glycol-co-poly (methacrylic acid) hydrogels and reported an increase in thermal stability of the polymer after the crosslinking and polymerization process [47].

### 3.4. DSC

**Figure 5:**
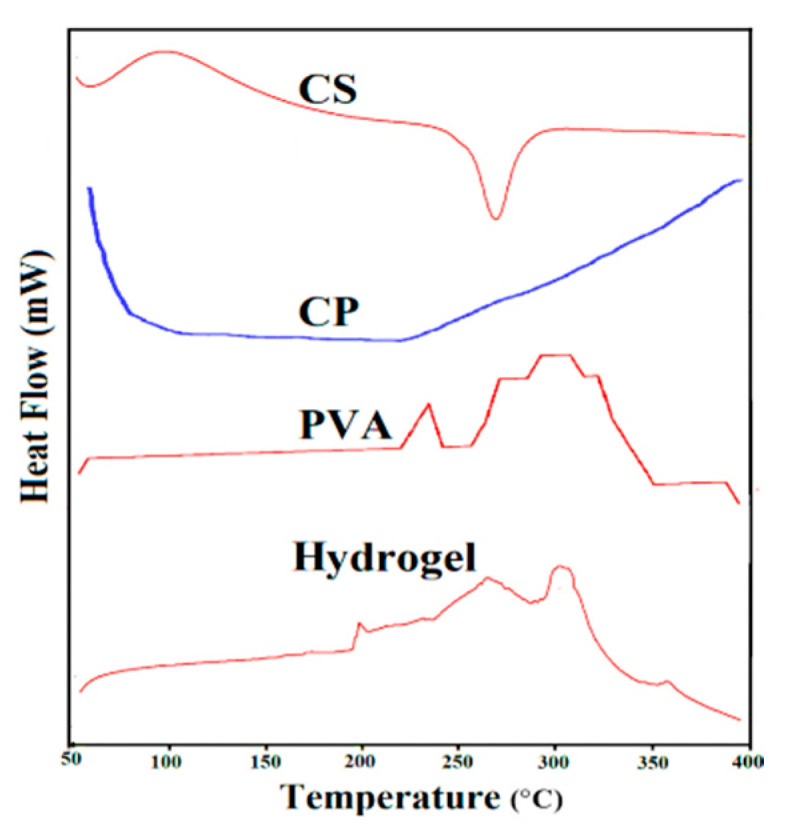
DSC of CS, CP, PVA, and CS/CP/PVAcPAa hydrogel.

The thermal stability of the polymers and fabricated hydrogel was also investigated by DSC, as indicated in Figure 5. The DSC of CS revealed an endothermic peak within a temperature range of 50–70 °C, which may be correlated with a possible change in polymeric chain or loss of volatile constituents. The degradation of CS was exhibited by a strong and broad exothermic peak within a temperature range of 80–150 °C. Another endothermic peak of CS was observed at 255 °C [48]. The DSC of CP depicted a sharp endothermic peak at 50–90 °C, which represented the initial moisture loss of the CP; subsequently, a glass transition temperature Tg peak was observed at 248 °C [39,49]. Similarly, the DSC of PVA revealed exothermic peaks at 245 and 298 °C, representing its decomposition. The glass transition temperature (Tg) of the PVA was observed at 53 °C. Aminabhavi and his co-workers reported the same peaks of PVA [50], which further supports our findings. Three exothermic peaks were depicted by the DSC of developed hydrogels at 200, 258, and 308 °C, demonstrating the exothermic peaks of CS and PVA moved from 70, 245, and 298 °C, respectively. The increase in the exothermic peaks of polymers in the developed hydrogel indicated greater constancy and thermal stability of the prepared hydrogel. Similarly, an endothermic peak was observed at 298 °C, representing the shifted endothermic peaks of CS at 255 to 298 °C of the prepared hydrogel. Thus, we can conclude from the discussion that the prepared hydrogel exhibited greater thermal stability as compared to unreacted polymers. Singh et al. (2019) synthesized antibiotic loaded hydrogel and reported high thermal stability of the prepared hydrogels as compared to their unreacted contents [51], which further supports our investigations.

### 3.5. X-ray Diffraction Studies

**Figure 6:**
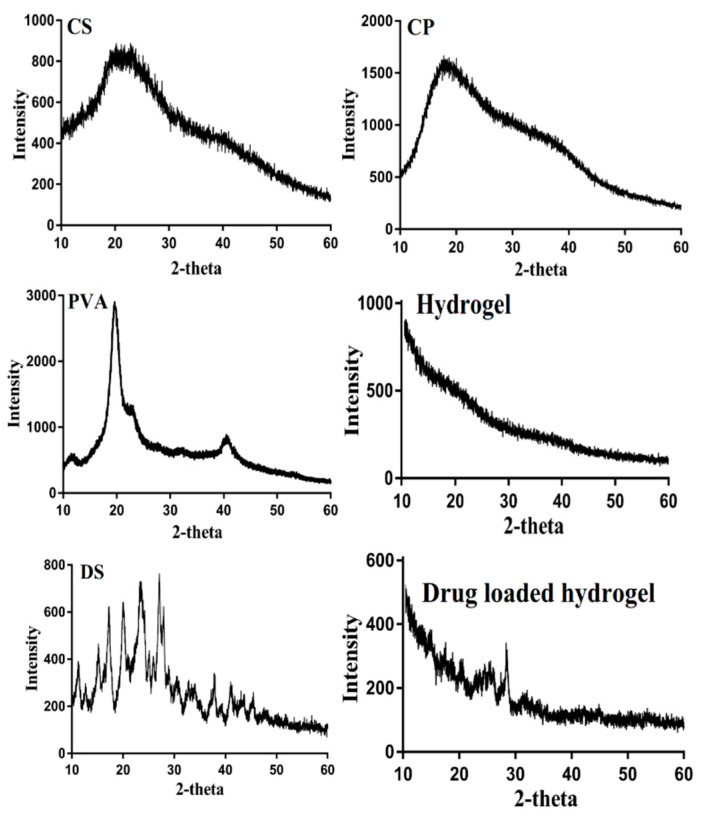
XRD of CS, CP, PVA, CS/CP/PVAcPAa hydrogel, DS, and drug-loaded CS/CP/PVAcPAa hydrogel.

The crystallinity of CS, CP, PVA, unloaded CS/CP/PVAcPAa hydrogel, DS, and drug-loaded Cp/PVA-g-PAa hydrogel was determined by XRD analysis as indicated in Figure 6. The XRD of CS revealed small crystalline peaks by 2θ = 22.62°, 27.43°, and 40.72°, while CP depicted its crystallinity by 2θ = 22.34°, 25.13°, and 38.10°, respectively. Similarly, PVA indicated its high intense crystalline peaks by 2θ = 19.41°, 21.92°, and 41.25°, respectively [52]. Due to successful crosslinking among hydrogel contents, the small intense and high crystalline peaks of CS, CP, and PVA were decreased to low intensity peaks, as observed in the XRD of unloaded CS/CP/PVAcPAa hydrogel. Lee and coworkers prepared polymeric networks of hydrogels and reported a decrease in the crystallinity of the reacted ingredients by the developed hydrogel [53]. Likewise, the XRD of DS indicated crystallinity by 2θ = 18.40°, 20.90°, 26.12°, 38.60°, and 41.04°, respectively. After the loading of DS by the formulated hydrogel, the crystalline peaks of DS were reduced to low intensity peaks, which demonstrated the amorphous nature of the CS/CP/PVAcPAa hydrogel. Comparing the XRD pattern of both unloaded and loaded hydrogel, a small change in the peaks position of the loaded hydrogels was observed due to the encapsulation of the DS, while the rest of the network of both unloaded and loaded hydrogel was the same. The PXRD of both unloaded and drug-loaded Cp/PVA-g-PAa hydrogel was almost the same [54].

### 3.6. SEM

**Figure 7:**
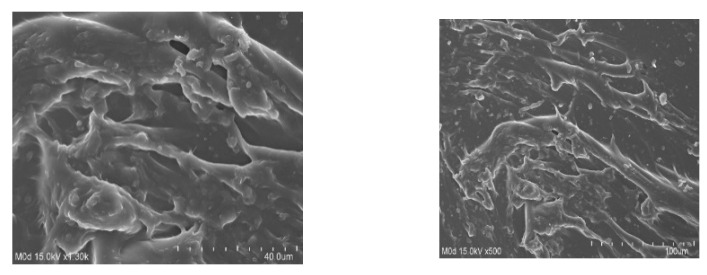
SEM of CS/CP/PVAcPAa hydrogel at different magnifications.

The microstructures and surface morphology of CS/CP/PVAcPAa hydrogel were investigated by SEM at different magnifications, as shown in Figure 7. Porosity and water retention capability of the hydrogel were evaluated by SEM. A rough surface with micro-spaces through which water absorption and drug entrapment occurred was revealed by SEM images. The water absorption occurred through the micropores of the hydrogel, and, as a result, swelling of hydrogel was exhibited. Due to swelling, entrapment of the drug occurred. Furthermore, DS was entrapped easily by the formulated hydrogel because of the irregular surface and presence of pores. Thus, due to the existence of pores, which led to high swelling and drug loading, the prepared hydrogel could act as a suitable drug carrier for the controlled drug delivery systems. Ali and co-workers prepared acrylic acid/ethylene glycol dimethacrylate-based hydrogel and reported a rough surface with micro-pores for absorption of water [55], which further supports our findings.

### 3.7. Sol–Gel Fraction

**Figure 8:**
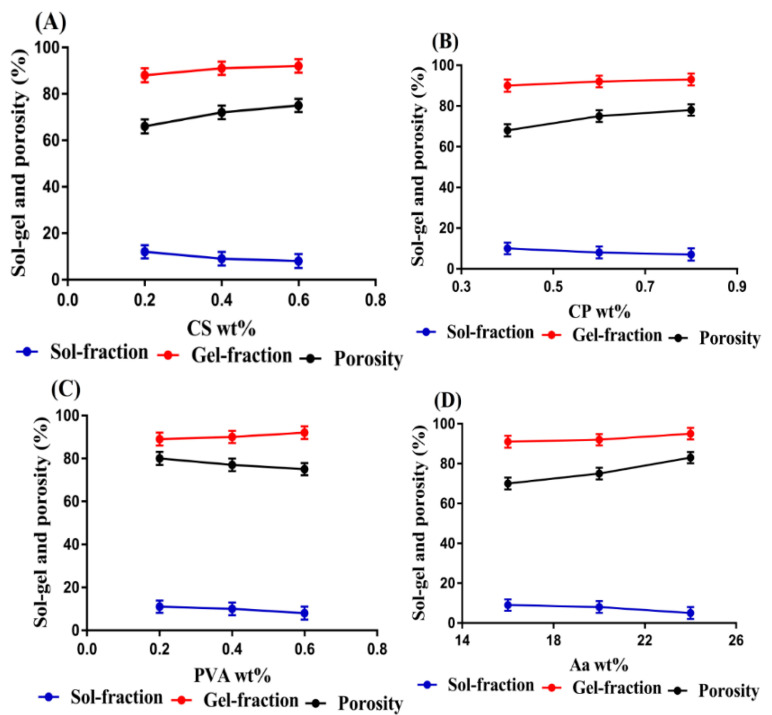
Effect of (**A**) CS, (**B**) CP, (**C**) PVA, and (**D**) Aa on sol–gel fraction and porosity of CS/CP/PVAcPAa hydrogel.

Sol–gel analysis was performed in order to know the soluble uncross-linked and insoluble crosslinked fraction of prepared hydrogels. Sol fraction, which is the soluble and uncross-linked fraction of the hydrogel, has an inverse relationship with the crosslinked insoluble gel fraction. Increase and decrease in gel fraction leads to decrease and increase in sol fraction. The sol and gel fractions were influenced by the use of various concentrations of CS, CP, PVA, and Aa, as shown in Figure 8. The sol fraction was decreased, whereas the gel fraction was increased with the enhancement in the concentrations of CS and CP. Due to high concentrations of CS and CP, a high number of free radicals were produced during the process of polymerization, which play an important role in crosslinking and grafting of contents. Thus, a fast polymerization reaction occurred among the hydrogel constituents due to the presence of a high number of free radicals, which led to a high gel and low sol fraction. Similarly, an increase in the gel and a decrease in the sol fraction was observed with the incorporation of high concentrations of PVA and Aa because high free radicals were available for the crosslinking and polymerization process, which led to high gel fraction and vice versa. Bashir and coworkers prepared Galantamine hydrobromide loaded hydrogels and reported an increase in gel and a decrease in sol fraction with the increase in the concentrations of hydrogel constituents [56].

### 3.8. Porosity Study

The main purpose of this study was to evaluate the penetration capability of a fluid through the fabricated hydrogels. Hence, the effects of various concentrations of CS, CP, PVA, and Aa on the pore size of the hydrogel were evaluated, as shown in Figure 8. Increase in porosity was observed with the increasing concentrations of CS, CP, and Aa. During the polymerization process, the viscosity of the mixture was increased due to the usage of high concentrations of CS, CP, and Aa. This viscous mixture restricted the evaporation of bubbles, due to which interconnected channels were produced, which led to an increase in the porosity of the hydrogels. Thus, an increase in porosity was observed with the incorporation of high concentrations of CS, CP, and Aa. Contrary to CS, CP, and Aa, a reduction in porosity was observed as the concentration of PVA was increased. A hard and bulk network of hydrogel was developed with the incorporation of high concentrations of PVA, which retarded the motility of the hydrogel networks, and, thus, a decrease in porosity was observed. A key role is played by porosity in swelling, loading, and release of the drug from the fabricated hydrogels. Generally, it is assumed that the higher the pore size of the hydrogel, the greater the swelling, loading, and release of the drug and vice versa [57].

### 3.9. Biodegradation Study

**Figure 9:**
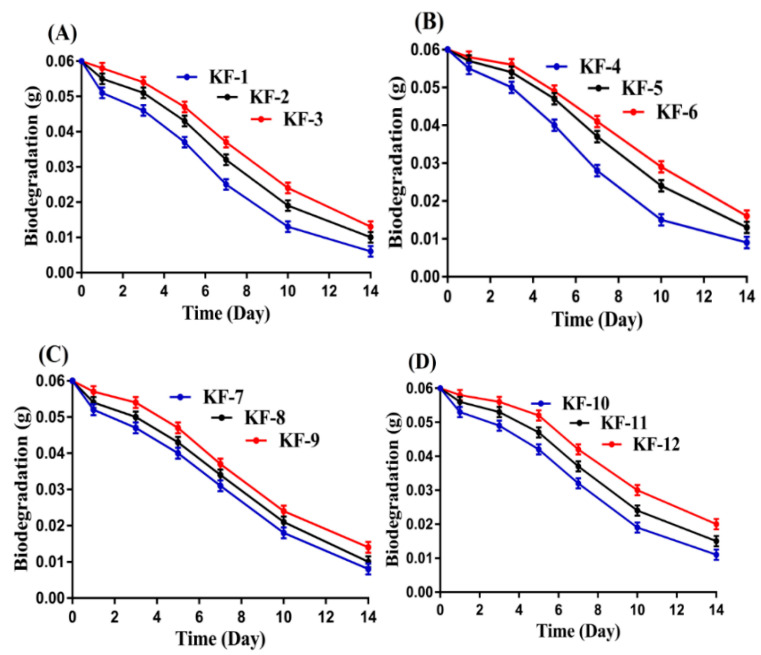
Effect of (**A**) CS (KF-1-3), (**B**) CP (KF-4-6), (**C**) PVA (KF-7-9), and (**D**) Aa (KF-10-12) on biodegradation of CS/CP/PVAcPAa hydrogel.

The degradation rate of the CS/CP/PVAcPAa hydrogel was determined by biodegradation study, as shown in Figure 9. The degradation rate of the prepared hydrogel was affected greatly by the different concentrations of the CS, CP, PVA, and Aa. The degradation of the developed hydrogel was found slow as the concentration of CS, CP, and PVA was increased. The possible reason may be correlated with the generation of free radicals, which led to high gelation, and, as a result, a strong crosslinked hydrogel network was formed. Thus, due to strong crosslinking among hydrogel contents, the bulk density of the hydrogel increased, due to which slow degradation of the hydrogel occurred. Similarly, incorporation of a high concentration of Aa with polymers contents led to a slow degradation of the hydrogel. Due to a high concentration of Aa, the polymerization reaction occurred very rapidly, and, as a result, high gelation was achieved. This crosslinking increased the mechanical strength of the hydrogel networks, and, thus, a slow degradation of the hydrogel was observed. Mohamed et al. (2015) prepared chitosan/PVA-based hydrogels and reported a slow degradation rate of the prepared hydrogel as the feed ratios of the hydrogel contents were increased [58].

### 3.10. Swelling Study

#### 3.10.1. Effect of pH

**Figure 10:**
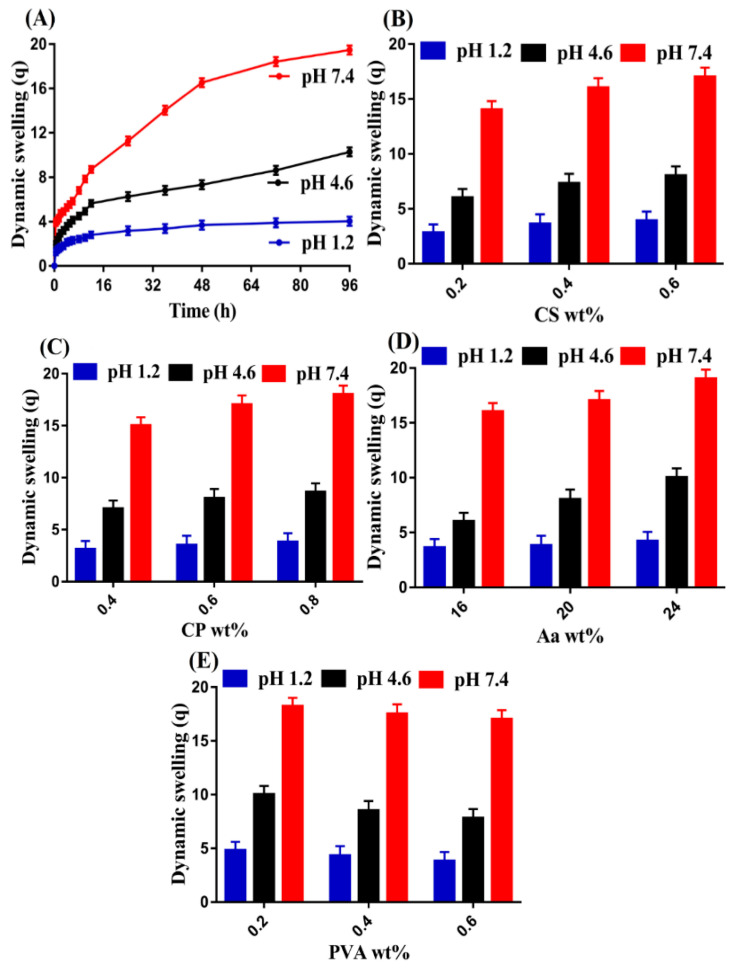
Effect of (**A**) pH, (**B**) CS, (**C**) CP, (**D**) Aa, and (**E**) PVA on dynamic swelling of CS/CP/PVAcPAa hydrogel.

Swelling study was conducted with the purpose to determine the pH-responsive nature of the developed hydrogel in three different pH values, i.e., pH 1.2, 4.6, and 7.4, as shown in Figure 10A. The fabricated hydrogel was affected highly by the pH of the medium as low swelling was observed at pH 1.2 as compared to pH 4.6 and 7.4, respectively. The low swelling at pH 1.2 yet high swelling at pH 4.6 and especially at pH 7.4 was due to the protonation and deprotonation of the functional groups of the pH-sensitive polymers (i.e., CS, CP, and PVA) and monomer Aa. CS, CP, and Aa contain COOH functional groups, which protonate at a low pH and deprotonate at high pH values due to their pKa values. Similarly, PVA contains OH functional groups, which protonate and deprotonate at low and high pH values. At pH 1.2, the functional groups of polymers and monomer formed conjugates with counter ions through strong hydrogen bonding, due to which the charge density of the same groups was decreased. Therefore, low swelling was achieved at pH 1.2. On the other hand, the change in the pH of the medium from 1.2 to 4.6 and 7.4 led to high charge density of the same groups, which resulted in generation of strong electrostatic repulsive forces. These forces caused repulsion of the same charged functional groups, and, thus, high swelling was observed at pH 4.6 and especially at pH 7.4 [59].

#### 3.10.2. Effect of CS/CP/PVA/ and Aa

As with pH, swelling of the hydrogel was also influenced by the incorporation of various concentrations of polymers and monomer. An increase in swelling was observed with the enhancement of the concentrations of CS, CP, and Aa, as shown in Figure 10B–D. Due to the presence of COOH, SO_3_, and OH groups of CS, as well as COOH groups of CP and Aa, the hydrophilicity of the hydrogel networks was increased, which caused high swelling of the hydrogel. Thus, an increase in swelling was exhibited as the concentration of CS, CP, and Aa was increased and vice versa [60,61,62,63]. Contrary to CS, CP, and Aa, a high crosslinked hard network of the hydrogel was developed with the incorporation of a high concentration of PVA (Figure 10E), which retards the flexibility of the prepared hydrogel. Therefore, a decline in swelling was observed as the concentration of PVA was increased [64].

### 3.11. Polymer Volume Fraction

The fraction of polymer in the swollen state of the prepared hydrogel was evaluated by polymer volume fraction study in three altered pH values of 1.2, 4.6, and 7.4, as indicated in Table 2. At pH 1.2, a greater polymer volume fraction was achieved, while, at pH 4.6 and 7.4, a low polymer volume fraction was obtained. The polymer volume fraction of the developed hydrogel was influenced by the various concentrations of the incorporated polymers and monomer contents. A reduction in polymer volume was observed with the increase in the concentrations of CS, CP, and Aa, which may be related with the maximum swelling of the developed hydrogel due to the usage of high concentrations of CS, CP, and Aa. On other hand, the polymer volume fraction was increased as the concentration of PVA was increased. Due to the inverse relation between the swelling and polymer volume fraction, a decrease in one content leads to an increase in the other and vice versa. The low polymer volume fraction at pH 4.6 and 7.4 and high at pH 1.2 represented low swelling of the prepared hydrogel at low pH value compared to high pH values [32].

### 3.12. Drug Loading

The drug loading by the fabricated hydrogel is totally dependent on swelling, which directly relies on the porosity of the hydrogel. In other words, the higher the porosity, the greater the swelling, and, thus, the higher the drug loading by the prepared hydrogel and vice versa. As with porosity and swelling, drug loading was also affected by the various concentrations of incorporated polymers and monomer, as shown in Table 2. Due to maximum swelling, high drug loading was observed with the enhancement in CS, CP, and Aa concentrations. Meanwhile, in the case of high concentration of PVA, drug loading was decreased due to the formation of a high bulk network of hydrogel, which restricted the water penetration into the hydrogel network. The pore size of the developed hydrogel was decreased due to high concentration of PVA, and, thus, low swelling was exhibited, which led to low drug loading [65].

### 3.13. Drug Release Studies

#### 3.13.1. Effect of pH

**Figure 11:**
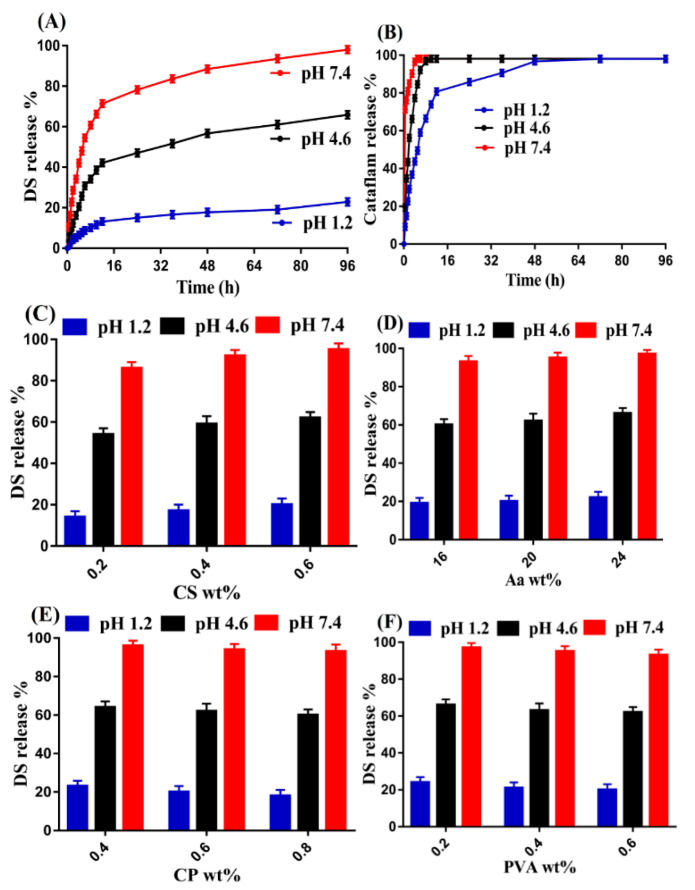
Effect of pH on drug release from (**A**) CS/CP/PVAcPAa hydrogel, (**B**) Cataflam, and effect of (**C**) CS, (**D**) Aa, (**E**) CP, and (**F**) PVA on drug release from CS/CP/PVAcPAa hydrogel.

A pH-dependent drug release was observed by the fabricated hydrogels in three different pH values, as shown in Figure 11A. A high drug release was observed at pH 4.6 and 7.4, while, in the case of pH 1.2, almost low drug release was perceived, which demonstrated the pH-responsive nature of the developed hydrogels. Due to the deprotonation of functional groups of CS, CP, PVA, and Aa at pH 4.6 and 7.4, high charge density was generated, and, as a result, strong electrostatic repulsive forces were produced, which caused repulsion of the same charged groups. Thus, an increase in swelling and drug release was perceived at pH 4.6 and 7.4. Unlikely, due to protonation of functional groups of the polymers and monomer at low pH value, almost low drug release was detected at pH 1.2 [66]. As with the developed hydrogel, drug release studies were conducted for commercial product Cataflam in pH 1.2, 4.6, and 7.4, as shown in Figure 11B. A drug release of 90% and 96% was observed within the initial 5 h and 3 h at pH 4.6 and 7.4, respectively. Similarly, 80% release of drug was detected at pH 1.2 within initial 12 h. Comparing the percent drug release of the developed hydrogel with Cataflam, we can demonstrate that drug release was sustained for a prolonged time by the prepared hydrogels as compared to the commercial product. Thus, prepared hydrogel networks could be considered as a suitable drug carrier for the controlled delivery of drugs.

#### 3.13.2. Effect of CS/CP/PVA/ and Aa

Drug release was affected by the various concentrations of incorporated hydrogel contents. An increase in the release of the drug from the fabricated hydrogel was observed as the concentration of CS and Aa increased (Figure 11C,D). The reason may be the hydrophilic nature of the CS and Aa due to the presence of COOH, SO3, and OH groups, which generated highly as the concentration of CS and Aa increased. These all resulted in an increase in the hydrophilicity of the hydrogels, and, thus, increase in drug release was achieved [67,68]. Contrary to CS and Aa, a decline in the release of drug was perceived with the increasing concentration of CP and PVA (Figure 11E,F). The viscosity of the hydrogel network increased due to the loading of greater drug with high incorporated CP concentration, which slowly and gradually released the drug and vice versa [69]. Similarly, high concentration of PVA restricted enough penetration of water into the hydrogel network, due to which low swelling, loading, and almost low release of the drug were observed [70].

### 3.14. Kinetic Modeling

Different kinetic models were used, such as zero-order, first order, Higuchi, and Korsmeyer–Peppas.

The release mechanism of the drug from the prepared hydrogels was investigated by fitting the release data on kinetic models. Zero-order failed to discuss the release order of the drug from the developed hydrogels, whereas first order of kinetics was followed by all formulations due to the closeness of their “r” values to 1. Further, “r” values of the Higuchi model demonstrated a controlled drug release from the prepared polymeric networks of hydrogel. The Korsmeyer–Peppas model explained the “n” diffusion exponent, which defines the mechanism of drug release from the prepared hydrogels, and “n” value demonstrated the type of diffusion. If 0.45 ≤ n, then diffusion will be Fickian diffusion, and, if 0.45 ≤ n ≤ 0.89, then diffusion will be non-Fickian (anomalous) transport analogous to coupled diffusion/polymer relaxation [71]. Further, “n” values of all formulations of the fabricated hydrogels are shown in Table 3, which were found within the range of 0.5012–0.6187, representing a non-Fickian diffusion [72].

### 3.15. Cytotoxicity Study

**Figure 12:**
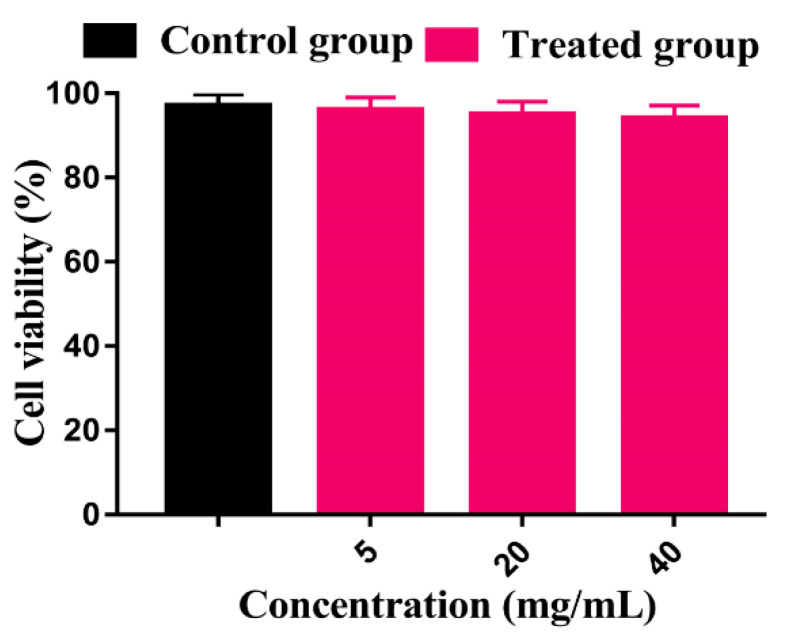
Cytotoxic testing of control and treated groups (CS/CP/PVAcPAa hydrogel).

The toxicity of the prepared hydrogel was investigated by cytotoxicity study. Cytotoxicity is the key study conducted for the detection of toxic effects of the proposed materials used in different pharmaceutical and biomedical fields. In the present investigation, viability of human colon cancer cells (T84) was examined on prepared hydrogels by the MTT cytotoxicity assay. The literature demonstrated that, if the cell viability of a material is greater than 70% as compared to the blank sample, then the material is non-cytotoxic. The prepared hydrogels were found safe for clinical purpose as no cytotoxic effect was shown by the prepared hydrogels at different concentrations because the cell viability for the fabricated hydrogels was found to be greater than 94% after incubation for 24 h, as indicated in Figure 12. Therefore, we can conclude that the developed hydrogels are safe and can be employed for clinical purposes in the near future [73].

## 4. Conclusions

A new polymeric pH-responsive hydrogel based on the CS/CP/PVA polymerized with Aa was prepared by the free radical polymerization technique for the oral controlled delivery of DS. The polymeric hydrogels presented various gel fractions, porosity, swelling, loading, and drug release depending on the concentration of CS, CP, PVA, and Aa. Increases in the gel fraction, porosity, swelling, loading, and drug release were observed with the increasing concentration of CS and Aa. PVA increased the gel fraction, while other parameters were decreased with its high concentration. CP decreased the drug release only, while the effects on other studies were same as like CS and Aa with its increased concentrations. Due to the usage of pH-sensitive polymers and monomer, the pH-sensitivity of the hydrogel networks was increased, and, thus, the maximum swelling and drug release were observed at pH 4.6 and 7.4 as compared to pH 1.2. Similarly, the degradation rate was slowed with the increment in the concentrations of polymers and monomer because a compatible crosslinked network was formed with the usage of high incorporated CS, CP, PVA, and Aa content. The cytotoxicity study revealed no toxic effect of the prepared hydrogels on human colon cancer cells (T84); thus, it was found to be safe and could be used on a clinical basis in the future as well. Likewise, FTIR confirmed the presence of the functional groups of the reagents in the prepared hydrogel, which indicated the development of a new polymeric network of hydrogel. TGA and DSC indicated high thermal stability of the prepared hydrogel as compared to its pure polymers. XRD presented a decrease in the crystallinity of the drug and polymers. SEM confirmed a hard surface with micropores through which penetration of water occurred. Conclusively, these pH-responsive hydrogels not only protect the drug from the acidic environment of the stomach but also protect the stomach from the adverse effects of the drug. Therefore, the prepared hydrogels could lead to a successful application for oral controlled drug delivery.

## Figures and Tables

**Table 1:** Feed ratio scheme for formulation of CS/CP/PVAcPAa hydrogels.

F. Code	Polymer CS g/100 g	Polymer CP g/100 g	Polymer PVA g/100 g	Monomer Aa g/100 g
KF-1	0.2	0.8	0.6	20
KF-2	0.4	0.8	0.6	20
KF-3	0.6	0.8	0.6	20
KF-4	0.4	0.4	0.6	20
KF-5	0.4	0.6	0.6	20
KF-6	0.4	0.8	0.6	20
KF-7	0.4	0.8	0.2	20
KF-8	0.4	0.8	0.4	20
KF-9	0.4	0.8	0.6	20
KF-10	0.4	0.8	0.6	16
KF-11	0.4	0.8	0.6	20
KF-12	0.4	0.8	0.6	24

A constant concentration of initiator APS (0.4 g/100 g) and cross-linker EGDMA (0.8 g/100 g) was used in all formulations.

**Table 2:** Polymer volume fraction and drug loading of CS/CP/PVAcPAa hydrogels.

Formulation Code	Polymer Volume Fraction			Drug-Loaded (mg)/450 mg of Dry Gel	
	pH1.2	pH 4.6	pH 7.4	Weight Method	Extraction Method
KF-1	0.303	0.166	0.071	152.2 ± 0.3	150.6 ± 0.2
KF-2	0.277	0.136	0.062	178.4 ± 0.2	177.2 ± 0.4
KF-3	0.256	0.125	0.058	194.1 ± 0.4	193.2 ± 0.3
KF-4	0.318	0.174	0.076	143.3 ± 0.6	142.1 ± 0.5
KF-5	0.284	0.146	0.067	164.6 ± 0.1	162.8 ± 0.1
KF-6	0.277	0.136	0.062	178.4 ± 0.2	177.2 ± 0.4
KF-7	0.208	0.105	0.053	205.1 ± 0.6	203.4 ± 0.1
KF-8	0.250	0.130	0.059	187.2 ± 0.5	185.3 ± 0.3
KF-9	0.277	0.136	0.062	178.4 ± 0.2	177.2 ± 0.4
KF-10	0.312	0.172	0.074	146.1 ± 0.4	144.4 ± 0.5
KF-11	0.277	0.136	0.062	178.4 ± 0.2	177.2 ± 0.2
KF-12	0.225	0.096	0.046	219.6 ± 0.1	209.1 ± 0.4

**Table 3:** Kinetic modeling of DS release from CS/CP/PVAcPAa hydrogels.

F. Code	Zero-Order r^2^	First Order r^2^	Higuchi r^2^	Korsmeyer–Peppas	
				r^2^	n
KF-1	0.9321	0.9943	0.9860	0.9132	0.5278
KF-2	0.9672	0.9987	0.9665	0.9240	0.5460
KF-3	0.9080	0.9812	0.9792	0.9429	0.5193
KF-4	0.9731	0.9889	0.9812	0.9782	0.5012
KF-5	0.9187	0.9973	0.9932	0.9672	0.5567
KF-6	0.9672	0.9987	0.9665	0.9240	0.5460
KF-7	0.9874	0.9894	0.9710	0.9621	0.6064
KF-8	0.9656	0.9950	0.9903	0.9893	0.5864
KF-9	0.9672	0.9987	0.9665	0.9240	0.5460
KF-10	0.9939	0.9991	0.9845	0.9782	0.6187
KF-11	0.9672	0.9987	0.9665	0.9240	0.5460
KF-12	0.9757	0.9884	0.9863	0.9824	0.5983
